# Session Intro: ARTIFICIAL INTELLIGENCE FOR ENHANCING CLINICAL MEDICINE

**Published:** 2020

**Authors:** Roxana Daneshjou, Lukasz Kidzinski, Olga Afanasiev, Jonathan H. Chen

**Affiliations:** Department of Dermatology, Stanford School of Medicine, Redwood City, CA 94063, United States; Department of Bioengineering, Stanford School of Medicine, Stanford, CA 94305, United States; Palo Alto Medical Foundation, 401 Old San Francisco Roud, Sunnyvale, CA 94086; Stanford Department of Medicine, Center for Biomedical Informatics Research and Division of Hospital Medicine, Stanford School of Medicine, Stanford, CA 94305, USA

**Keywords:** artificial intelligence, machine learning, bioinformatics, augmented clinical decision making

## Abstract

Machine learning and deep learning have revolutionized our ability to analyze and find patterns in multi-dimensional and intricate datasets. As such, these methods have the ability to help us decipher the large volume of data generated within healthcare. These tools hold the promise of enhancing patient care through several modalities, including clinical decision support, monitoring tools, image interpretation, and triaging capabilities. For the 2020 Pacific Symposium on Biocomputing’s session on Artificial Intelligence for Enhancing Clinical Medicine, we highlight novel research on the application of artificial intelligence to solve problems within the field of medicine.

## 1. Introduction

Artificial intelligence aims to create computer programs that can abstractly reason and make generalizations about the world.^1^ While general artificial intelligence remains elusive, we observe continued advances and applications in machine learning, the science of creating algorithms that improve in performance with increasing data and experience.^1^ Deep learning, a subset of machine learning that uses multi-layer networks, has particularly gained popularity in the past several years for solving complex tasks with large amounts of data. As the emerging potential of such technology advances, researchers have been fascinated with the idea of harnessing artificial intelligence for accomplishing tasks in biomedical research and clinical medicine, an idea first proposed in the 1970s.^2^

Medicine is now ripe for the use of such technology with the rise in large volumes of multi-dimensional data which has grown beyond the limitations of humans to interpret.^3^ A wide range of studies applying artificial intelligence to medicine have been published, including algorithms to predict radiology imaging findings, dermatologic diagnoses, and patient outcomes from electronic medical record data.^3^ Here we highlight cutting edge research in the field selected for presentation at the 2020 Pacific Symposium on Biocomputing session on “Artificial Intelligence for Enhancing Clinical Medicine.”

## 2. Novel Research Applying Artificial Intelligence to Clinical Medicine

### 2.1. Artificial intelligence for predicting patient outcomes

Healthcare data is intricate and multi-modal – on a given day, a physician may need to synthesize prior encounter notes, patient vitals, the patient’s physical exam, labs, imaging data, and other measures in order to make clinical decisions and predict patient outcomes. For complicated patients who have interacted often with the healthcare system, the amount of information is compounded. A recurring theme among artificial intelligence papers in medicine is the use artificial intelligence to evaluate the large volume of clinical data and to make predictions about patient outcomes from disease states to treatment outcomes.

Understanding the relationship between symptoms and diagnosis is important for the diagnostic process. A health knowledge graph is a representation of the connections between symptoms and diseases and can help with diagnosis prediction tasks. Chen and Agrawal et al present results evaluating health knowledge graphs and looking at what features are important for accurate diagnosis prediction.^4^ They identify major sources of error including unmeasured confounders and sample size and discuss new methods of robust medical knowledge extraction from electronic health records.^4^

Diagnosing a disease is not always straightforward; some disease states require multiple tests and longitudinal evaluation before a diagnosis is established. Artificial intelligence may play a role in helping diagnose a disease state sooner by learning predictive patterns that humans may miss. For example, Alzheimer’s disease is a neurodegenerative disorder marked with progressive loss of cognitive ability, and researchers hope to better predict an early diagnosis based on patient data.^5^ Lodewijk Brand and colleagues used a tensor-based joint classification and regression model to make Alzheimer’s disease diagnostic predictions based on longitudinal multi-modal data that included imaging data, cognitive testing, and genetic data generated by the Alzheimer’s Disease Neuroimaging Initiative.^5^ The use of longitudinal, multi-modal data for model creation represents an important requirement for the application of artificial intelligence to medicine, as such data is representative of real world use cases.

In the acute care setting such as in the intensive care unit, predicting which patients are improving and which patients require increased vigilance could help with informing clinical care. Yu et al. explore techniques from Natural Language Processing to analyze Electronic Health Records (EHR) for predicting mortality risk in ICU patients, outperforming existing severity scoring systems (SAPS-II).^6^ They use bag-of-words representation of EHR to deal with missing data, Latent Semantic Analysis to reduce the dimensionality, and bidirectional Long Short-Term Memory (LSTM) networks as regression models.^6^ They conjecture that superior performance is due to the bidirectional nature of their model.^6^

Predicting future disease and healthcare costs could allow targeted preventative care for at risk patients. To that end, Xianlong Zeng et al. developed a multilevel self-attention model to account for the dependencies and temporal relationships between medical diagnoses as represented by medical codes.^7^ They applied their model to two large real-world datasets to predict disease and healthcare costs with improved performance compared to previous models.^7^ Along the same lines, Haoran Zhang and colleagues were also looking to predict which patients were at risk of becoming high cost users; however, their model used free form text data from electronic medical records of family care practices in Ontario.^8^ As much of medical data is free form through unstructured medical notes, building models that can handle this type of data has the potential for many applications.

Predicting treatment outcomes is important for patient care, since these predictions can inform clinicians trying to decide whether a treatment course should continue or be changed. With treatment resistant major depressive disorder being treated with deep brain stimulation (DBS), there are no objective measures for treatment outcome; rather, a clinician administered measure is used based on the patient’s self-reported symptoms.^9^ Prediction using this clinician administered measure is further complicated, as patients often have a non-linear course on their way to improvement.^9^ However, recent research has suggested that facial expressions and other psychomotor attributes may be able to help predict DBS response in patients with major depressive disorder.^9^ In their paper, Harati and colleagues leveraged a joint state-estimation and temporal difference learning approach to be able to model patients’ treatment response from audio-visual features extracted from weekly video recordings taken during DBS treatment.^9^ Their results hold promise for the development of an objective measure for predicting DBS efficacy in patients with major depressive disorder.^9^

### 2.2. Artificial intelligence for improved insight into disease pathogenesis and features

Human disease is complex and multifactorial. Artificial intelligence can help make sense of large multifactorial disease datasets and discover patterns that would not have been easily discerned otherwise.

In cancer, somatic genomic alterations (SGAs) cause disruption of the normal cellular pathways, which can lead to oncogenesis. Thus, elucidating the functional impact of SGAs can inform our understanding of cancer development. Tao et al. propose a genomic impact transformer (GIT) model—an encoder-decoder deep neural network architecture to achieve state-of-the-art dimensionality reduction of somatic genomic alterations (SGA) patterns.^10^ They apply the model to 4468 tumors profiled by the Cancer Genome Atlas (TCGA) project and find it outperforms existing models in capturing the relationships between SGAs and differentially expressed genes.^10^ Moreover, they find that the latent representation given by GIT is predictive of tumor status, survival time and drug response.^10^

In medicine, a “disease” entity may actually have many subtypes, with different features and outcomes. Identifying these subtypes and outcomes depends on having a large enough dataset and an ability to find patterns. Vandromme et al. present an automatic method for phenotyping patients with non-alcoholic fatty liver disease (NAFLD).^11^ In the cohort of 13,290 patients labeled as NAFLD in the electronic health record, they aggregated four types of features: demographic, disease-specific based on ICD/CPT codes, lab tests, and vital signs.^11^ Clustering techniques revealed 5 clusters which were clinically distinct and associated with different rates of death and disease occurrence.^11^

Understanding the microbiome signatures of disease is an area of ongoing research. To that end, Khan and Kelly constructed predictive models for identifying 19 diseases in 5643 samples of whole-community metagenomes.^12^ They compare random forests, convolutional neural networks, and a graph convolution architecture, which they developed for the purpose of this study. Their new technique outperforms other models, and they find interesting disease specific signatures that could form the basis for further study.^12^

## 3. Artificial intelligence for advancing medical workflows

An exciting opportunity for artificial intelligence in medicine is the automation of previously manual workflows.

For example, clinical trial recruitment often requires humans to help identify patients that are eligible for the trial. This can be a tedious process, and the manual nature of it can cause eligible patients to be missed. Chen and Kunder et al address the need to efficiently screen patients and their tumor profiles for appropriate clinical trials.^13^ Their algorithm screens and matches genetic tumor biomarkers with NCIMATCH and internal precision medicine clinical trials to provide an automated pipeline for feasible, accurate and effective trial accrual.^13^

Another example is the clinical interpretation of genetic data. As genome sequencing costs plummet, genetic testing has become more common in clinical cases that are suspected to have a genetic component. However, clinically interpreting a list of genetic mutations requires manual validation by trained biocurators. These curators will search the literature for evidence of a mutation’s pathogenicity, looking at prior clinical studies and functional experiments.^14^ Nie et al created LitGen, which uses semi-supervised deep learning to retrieve papers for the genetic variant being studied and to predict the relevant evidence provided by that paper.^14^ This tool, in the hands of curators, could improve the time spent on identifying evidence for clinically relevant genetic variants.^14^

## 4. Artificial intelligence for improving imaging

Imaging is an important diagnostic tool in medicine, and improving data processing can save time and money. Zhao et al. proposed a convolutional neural network architecture to predict a difference between the two channels in a Dual-Energy Computed Tomography (CT) scan only from the low-energy channel.^15^ This allows approximating the high-energy signal using only Single-Energy CT systems, which are more prevalent in clinical imaging than Dual-Energy CT systems.^15^ The proposed technique can simplify data acquisition, optimize scanning doses, and reduce noise levels in CT imaging.^15^

## 5. Conclusion

Artificial intelligence has, and will have, a wide range of applications in medicine. This ranges from predicting patient outcomes such as diagnoses and treatment efficacy todiscovering patterns in large databases to understanding disease pathogenesis. When medicine is filled with tedious, manual tasks, artificial intelligence can power automated workflows to expand access to high quality healthcare. While, further maturation of the field will require prospective validation of such technology in live clinical settings, the research highlighted here continues to provide us an optimistic view of the potential for such advancing science and technology to improve lives by enhancing clinical medicine.
